# A Novel Dry Treatment for Municipal Solid Waste Incineration Bottom Ash for the Reduction of Salts and Potential Toxic Elements

**DOI:** 10.3390/ma14113133

**Published:** 2021-06-07

**Authors:** Marco Abis, Martina Bruno, Franz-Georg Simon, Raul Grönholm, Michel Hoppe, Kerstin Kuchta, Silvia Fiore

**Affiliations:** 1 SRWM (Sustainable Resource and Waste Management), Hamburg University of Technology, 21079 Hamburg, Germany; kuchta@tuhh.de; 2 DIATI (Department of Engineering for Environment, Land and Infrastructures), Politecnico di Torino, 10129 Torino, Italy; martina.bruno@polito.it; 3 Bundesanstalt für Materialforschung und -Prüfung (BAM), 12200 Berlin, Germany; franz-georg.simon@bam.de; 4 Sysav Utveckling AB, 20025 Malmö, Sweden; Raul.Gronholm@sysav.se; 5 Heidemann Recycling GmbH, 28277 Bremen, Germany; m.hoppe@heidemann-recycling.de

**Keywords:** bottom ash, dry treatment, incineration, municipal solid waste, potential toxic elements, salts

## Abstract

The main obstacle to bottom ash (BA) being used as a recycling aggregate is the content of salts and potential toxic elements (PTEs), concentrated in a layer that coats BA particles. This work presents a dry treatment for the removal of salts and PTEs from BA particles. Two pilot-scale abrasion units (with/without the removal of the fine particles) were fed with different BA samples. The performance of the abrasion tests was assessed through the analyses of particle size and moisture, and that of the column leaching tests at solid-to-liquid ratios between 0.3 and 4. The results were: the particle-size distribution of the treated materials was homogeneous (25 wt % had dimensions <6.3 mm) and their moisture halved, as well as the electrical conductivity of the leachates. A significant decrease was observed in the leachates of the treated BA for sulphates (44%), chlorides (26%), and PTEs (53% Cr, 60% Cu and 8% Mo). The statistical analysis revealed good correlations between chloride and sulphate concentrations in the leachates with Ba, Cu, Mo, and Sr, illustrating the consistent behavior of the major and minor components of the layer surrounding BA particles. In conclusion, the tested process could be considered as promising for the improvement of BA valorization.

## 1. Introduction

The mining of mineral aggregates is the largest extractive sector in the EU, which, on its own, exceeds the amount of all the minerals produced [1]. Nevertheless, its End-of-Life (EoL) input rate was estimated to be only 8 wt %. Typical EoL materials used as aggregates are construction and demolition waste (CDW) and bottom ash (BA) from municipal solid waste incineration (MSWI). However, the full recycling potential of these waste flows have not been tapped yet due to the existing gap between market prices and extraction, processing, and transportation costs [2].

BA is the main by-product of MSWI and counts approximately 25 wt % of MSW input for thermal valorization [3,4]; 71 Mt of MSW incinerated in Europe in 2018 produced about 18 Mt of BA. Current full-scale material recovery technologies applied to BA mainly focus on the separation of metals, with the most valuable components being aluminum and copper [5,6]; this leaves the mineral fraction unexploited, and which is usually landfilled. The mineral fraction has been estimated at 85–90 wt % of BA [4], resulting in 15–16 Mt/y of materials in Europe that could potentially be recycled as aggregates. Several studies have recently explored recycling alternatives for the BA mineral fraction to be used as construction material, e.g., as the sub-base layer for asphalt roads [7], as the source of construction sand [8] and as substitute material for concrete production [9,10]. The recycling of the BA mineral fraction as a secondary aggregate holds the potential of enhancing the profitability of BA management and is fully consistent with EU policy on Circular Economy. Material recovery from BA could entail a significant improvement in the circularity of the management of resources, not only limiting the request for primary aggregates but more particularly reducing the amount of waste sent to the landfill. Despite the low commercial value of secondary mineral aggregates, potential savings from landfill fees could entail significant economic benefits and ensure the profitability of BA mineral-fraction management. The production of primary aggregates in Europe in 2018 was 2431 Mt, which was composed of sand, gravel, and crushed rocks [11]. Considering this mass, the mineral fraction of BA could potentially replace 0.7 wt % of primary aggregates produced in Europe. This value was consistent with a recent study [12], which estimated a 0.6% potential substitution rate. Nonetheless, since about 806 Mt of non-hazardous waste generated in Europe are currently disposed in landfills, the recycling of the BA mineral fraction as a secondary aggregate might divert circa 2 wt % of the waste stream directed to landfills.

However, the potential reuse of the BA mineral fraction as a secondary aggregate is hindered by the presence of Potential Toxic Elements (PTEs) [13], which have negative environmental impacts [14,15]. Among the PTEs, cadmium, chromium, and molybdenum were the most found in MSWI BA [16,17]. Most European countries set threshold limits for recycled aggregates due to PTEs and chloride and sulphate leaching. A detailed analysis of the different leaching tests applied to BA and related limits in Europe were presented in a recent study [12]. In this framework, special attention should be devoted to studying BA composition, not only to detect the mineral phases for further geochemical dissolution modelling, but also to map their occurrence and locations in the solids. X-ray diffraction analyses (XRD), energy dispersive X-ray spectroscopy (EDX), and Scanning Electron Microscopy (SEM) were some analytical techniques adopted for such investigations. The key result of those characterization studies [18] was that the presence of chloride and sulphate salts is mostly limited to the surface layer coating the coarser BA particles. This observation justified the strong drops in chloride concentration observed by different authors [5,14,19] while performing percolation leaching tests on BA after washout and dissolution treatments. Similarly, the presence of other sparingly soluble salts, such as calcium sulphate and their similar releasing mechanisms, also suggests their accumulation in a layer coating the coarser BA particles. Finally, the existence of several PTEs was linked with the presence of calcite (CaCO_3_), melilite, and iron oxides [16], and weathering products such as gypsum, ettringite, and zeolite have been proven to contain high amounts of PTEs.

**Figure 1:**
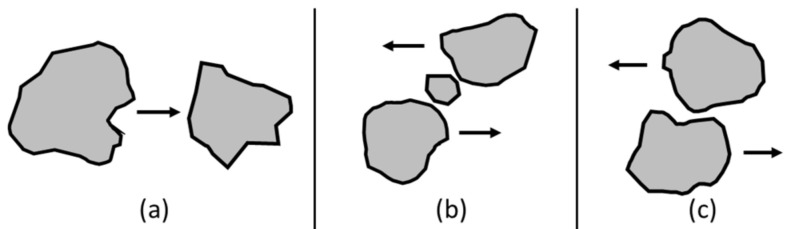
Grinding mechanisms: (**a**) compression, (**b**) chipping, (**c**) abrasion (adapted from Wills and Finch 2015 [27]).

Several efforts have been devoted to reducing the amounts of salts [20,21] and of the metals Zn, Cu, and Ni [22] from BA through the application of intensive washing processes, which were proven to be rather effective in the removal; however, run-off waters resulted as contaminated from the presence of heavy metals, chlorides, and sulphates [23]. Particularly, concern arose from the leaching of copper [24] and antimony [25], exceeding wastewater discharge limits. Therefore, wet processes aimed at reducing the leaching of salts and PTEs from the mineral fraction of BA, even if effective, still presented several critical downstream issues (e.g., wastewater treatment, sludge thickening, and disposal) in need of optimization. To our knowledge, no literature is available on dry treatment processes applied to the mineral fraction of BA; a dry process, if effective in reducing the release of salts and PTEs, could avoid any wastewater in need of further treatment. In this study, a novel dry treatment process was explored, based on the findings that most of the salts and PTEs released from BA are located on a thin, superficial layer coating coarser BA particles, which can be selectively removed by controlling the mutual abrasion of the particles in a tumbling mill. More precisely, while processing aggregates in a tumbling mill, the grinding of the material occurs. Grinding is driven by three main mechanisms [26]: impact (compression), chipping (attrition), and abrasion [27] when applied with normal, oblique, and parallel forces, respectively (Figure 1). The main objective of this study was to avoid and minimize the compression and chipping forces, and to maintain BA coarseness, while promoting the abrasion on the particles’ surfaces.

**Figure 2:**
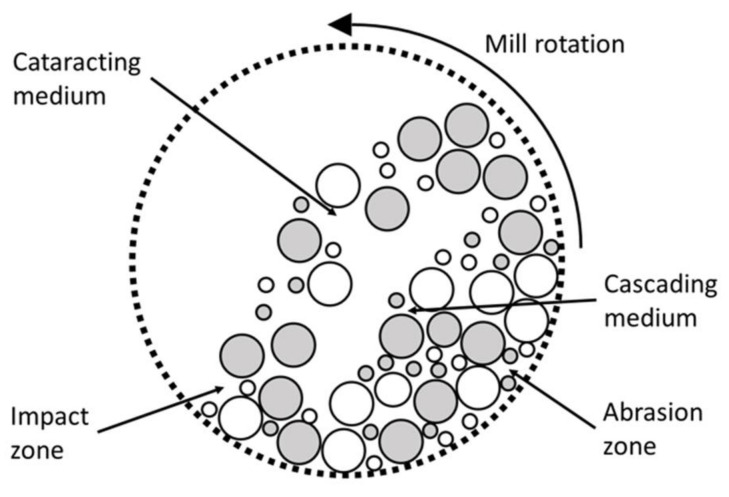
Aggregate motion in a tumbling mill (adapted from Ali et al., 2019 [28]; Wills 2016 [29]; Wills and Finch 2015 [27]).

The forces acting in a tumbling mill can be controlled by varying the operating rotational speed. High rotational speeds are associated with impact and compression forces, generated by particles that are cataracting and cascading (Figure 2). 

Alternatively, abrasion is promoted, operating at rotational speeds below 30% of the critical speed Cs, above which particles are only centrifuged without being ground [30]. For low rotational speeds, particles slide on the drum surface remaining in the abrasion zone (Table 1).

Consequently, the dry treatment process presented in this work did not rely simply on dissolution and mass transfer, which divert the contaminants to wastewater in need of further treatment (as in wet processes); instead, it took advantage of the natural abrasive behavior of BA particles, which scrub off each other’s external contamination. Moreover, abrasion allows BA particles to preserve their original size, and therefore remain suitable for recycling as secondary aggregates. The output streams of the treatment investigated in this study were a cleansed coarse fraction of inert material and a fine fraction easily removable by sieving. In particular, the intensity of the abrasion process was controlled to limit its effect on the superficial abrasion of the particles’ external layers, avoiding comminution and subsequently, particle-size reduction. This work was aimed at exploring the feasibility of a dry abrasion treatment for the reduction of the BA leaching potential for salts and contaminants, in the light of BA reuse as building materials. Furthermore, the effect of the applied treatment was assessed, comparing the leaching behaviors of treated and untreated BA samples. This approach was an advancement with regard to the current industrial trends, where there is no industrial alternative to the wet treatment of BA. To our knowledge, there are still no literature studies investigating abrasion processes applied to BA at the laboratory scale. The work in the present study was performed with batch experiments only within a laboratory setting. However, tumbling mills were applied in the comminution process of ores in continuous operation mode with throughputs in the range of thousands t/h [27]. In industrial processing plants, it is possible to increase the residence time in abrasion units, as well as install liners on the inner surface of rotating trommels in order to decrease the dimension of the processing unit and optimize its filling ratio.

## 2. Materials and Methods

### 2.1. Origin of the Samples

This research is part of the activities of the BASH-TREAT “Bottom ash treatment for an improved recovery of valuable fractions” project (ID-157), funded by ERA-NET Cofund in the 2017 call ERA-MIN2 “Research and Innovation Programme on Raw Materials to foster Circular Economy”. Specifically, this research considered the valorization of the mineral fraction derived from state-of-the-art BA treatment. The samples of BA mineral fractions were collected from two BA treatment plants located in Germany (Plant A) and Sweden (Plant B). The primary focus of both plants was the recovery of ferrous and non-ferrous metals. In these plants, BA is usually stored for 12 weeks before being processed, in order to decrease the moisture content to values compatible with the adopted technologies. Plant A discharges the fines (<2 mm) and produces two coarse mineral fractions (2–8 mm and 8–40 mm), which, for this study, were mixed in homogeneous proportions (38 wt % and 62 wt %, respectively) based on what happened in the plant. Plant B produces a mineral fraction with dimensions in the range 4–26 mm. For this study, 15 incremental samples were collected in each plant, directly at the conveyor belt discharge (sampling the entire section of the belt). The 15 samples were collected at regular intervals within one working day, and later merged into a single composite sample for each plant.

For the abrasion experiments, the mixture 2–40 mm for Plant A and the 4–26 mm fraction for Plant B were further investigated. The fines (0–2 mm for Plant A, 0–4 mm for Plant B) were not included in the abrasion tests since these fractions were not relevant for their potential reuse as mineral aggregates, and due to the presence of contaminants in high concentrations.

### 2.2. Abrasion Tests

The samples 2–40 mm (from Plant A) and 4–26 mm (from Plant B) underwent the abrasion tests. Two different pilot-scale rotating units were operated with increasing processing times to establish intense abrasion forces. Firstly, an initial abrasion test was performed for 240 min using a concrete mixer with an inner radius of 50 cm and a height of 60 cm. The use of a concrete mixer did not allow the removal of fine particles, while minimal losses of fugitive dust from the main opening occurred. Hence, a second abrasion unit was developed, including a sieving device able to remove the fine materials gradually produced. A cylindrical sieving drum (Scheppach RS 400), was coated with a 2 mm mesh stainless steel grate. The fines produced were collected directly at the bottom of the cylindrical drum for further analysis and characterization. The feedstock (12–14 kg) and the rotational speed (set at 42 rpm) were chosen to avoid the cascading of particles. The abrasion process was investigated by varying the abrasion time (60 and 120 min) (Table 2). For all the experiments, the mass and moisture of the samples before and after abrasion were evaluated in order to estimate the production of fines, loss of materials (fugitive dust), and water content. No grinding aids (e.g., steel balls or rods) were added in any of the experiments in order to achieve an autogenous grinding regime [31].

### 2.3. Leaching Tests

Column leaching tests were performed to evaluate the cumulated release of salts and PTEs before and after abrasion, at liquid to solid (L/S) ratio values equal to 0.3, 1, 2, 3, and 4.0 L/kg. For each sample, the tests were performed in triplicates. The columns (40 cm height, 10 mm internal diameter) were packed with undried samples, and quartz sand was placed on the top and bottom layers of the column (2 mm thickness, grain size: 0.7–1.2 mm). After the saturation of the column, percolation speed was increased according to DIN 19528 [32]. Approximately 3.5–4 kg of the sample was used for each column. The leachates were collected in closed glass bottles for further analyses. For each element, the released amount E_i_ at any L/S value was calculated (Equation (1)):

E_i_ = (c_i_ × V_i_)/m_d_ (mg/kg) (1)

where i is the index of the eluted fraction (0.3, 1, 2, 3, and 4 L/kg); c_i_ is the concentration (in mg/L) of the respective element in the leachate volume V_i_ (in litres); m_d_ (in kg) is the dry mass of the sample in the column. The cumulative leached amount U (with U_L/S_ = ΣE_i_, in mg/kg) for each element was given at L/S values of 2 and 4 L/kg (Equations (2) and (3)).

U_L/S = 2_ = E_0.3_ + E_1_ +E_2_ (2)

U_L/S = 4_ = E_0.3_ + E_1_ +E_2_ + E_3_ + E_4_ (3)

### 2.4. Analytical Procedures

The moisture content was evaluated on each BA mineral fraction sample by drying batches of materials in porcelain containers at 105 °C, according to DIN EN ISO 17829-1 [33]. The particle size distribution analysis was performed on ash samples before and after the abrasion experiments using a vibratory sieve shaker (Retsch AS 300 control), according to DIN EN 933-1 [34], without any wet removal of the fines. 

The procedures adopted to analyze the leachates from the percolation leaching tests were as follows: Immediately after collecting each sample, the electrical conductivity (EC) was measured through a WTW Multi 3320 portable probe. Chlorides were analyzed by titration, following the DIN 38405-1 [35]. Dissolved organic carbon (DOC) was measured through a Multi N/C 2000 analyzer from Analytik Jena AG. Sulphates were analyzed through a Hach DR3900 spectrophotometer. For major elements and PTEs analyses (Ca, K, Na, Al, B, Ba, Co, Cr, Cu, Fe, Li, Mg, Mn, Mo, Ni, Sr, Ti, V, Zr), a part of the leachates was acidified with a small addition (1:100) of 65% nitric acid and analyzed through an ICP-OES Agilent 5100 spectrometer. All analyses were performed in triplicates.

### 2.5. Data Analysis

The experimental results derived from the analyses of the leachates that came from the column leaching tests were analyzed though a Pearson correlation analysis (two-tailed, 95% confidence) by means of the SPSS Statistics software. The Pearson correlation considered a significance level of α ≤ 0.05.

## 3. Results

### 3.1. Physical Characterization

**Figure 3:**
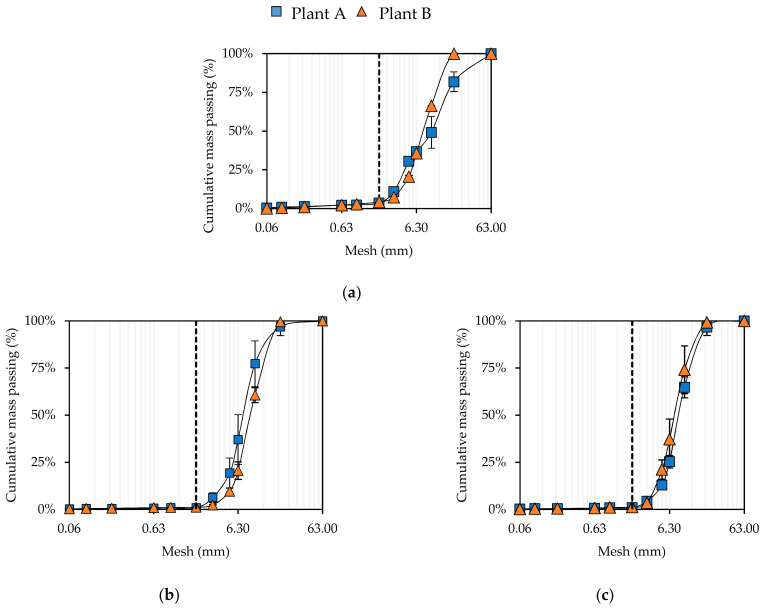
Particle size distribution of (**a**) the raw samples from Plant A and Plant B, of (**b**) samples treated for 60 min and of (**c**) samples treated for 120 min. Dotted line is mesh = 2 mm.

The particle-size distributions of the BA mineral fraction samples (Figure 3a) were rather different; the samples collected at Plant A presented lower amounts of coarse particles compared to samples from Plant B, although obtaining a greater standard deviation. Considering the particle-size distribution after the abrasion tests (Figure 3b,c), it is possible to observe that, despite the differences between the raw samples, the particle size distribution of the treated samples assumed a coherent behavior, following a narrow bundle where about 25% of the particles had a dimension of below 6.3 mm. For Plant A the additional fine fraction produced was 25% and 38% of the input after 60 min and 120 min abrasion, respectively. For Plant B, the fines produced were 12% and 16% after 60 min and 120 min abrasion, respectively. The significant difference between the processes performed in the two plants was explained by the presence of near-size particles in the Plant A sample, in the fraction proximate to 2 mm (Plant A range: 2–40 mm); however, these were absent in Plant B (range: 4–26 mm). The future optimization of the abrasion process should consider the use of a smaller screen mesh to prevent the loss of particles slightly bigger than 2 mm, together with the abrasion products. Alternatively, the feedstock must be screened to avoid the presence of near-size particles (as occurred naturally for the Plant B material).

**Figure 4:**
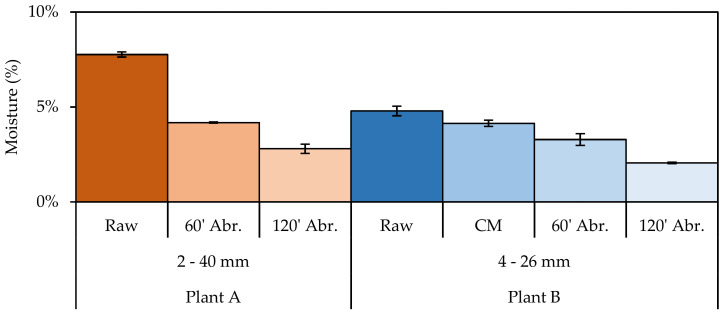
Moisture content of the raw samples from Plant A and Plant B, of the samples treated for 60 and 120 min abrasion (Abr.) time, and those treated with the concrete mixer (only Plant B).

The moisture content decreased drastically in the samples processed with the abrasion unit with removal of the fine particles. Considering the samples from Plant A, the initial moisture was 7.8%, decreased to 4.2% after 60 min of abrasion, and further diminished to 2.8% after 120 min of abrasion. Similarly, for the samples from Plant B, the initial moisture was 4.8%, while the material abraded with the concrete mixer showed a moisture of 4.1%. The moisture was further decreased to 3.3% and 2.1% after 60 min and 120 min abrasion times, respectively (Figure 4). In both samples a limited decrease in the d_50_ after abrasion can be observed: in plant A, d_50_ decreased from 10.25 mm to 8.62 mm after 120 min of abrasion (−16%), while in plant B, d_50_ decreased from 8.03 mm to 7.58 mm after the same abrasion time (−6%).

### 3.2. Leaching Tests

The performance of the investigated abrasion process was estimated by comparing the results of column leaching tests prior to and after treatment, and by analyzing the release of major salts and PTEs.

#### 3.2.1. Release of Major Salts

The purpose of the developed process was to promote the mutual friction between the ash particles. The abrasion, combined with the prompt removal of progressively generated fines and dust, led to a qualitative improvement of the particles’ aspect and leaching potential. The intense abrasive forces in the abrasion units were capable of mechanically removing the outer shells of the particles by both smoothing and sanding the ash surfaces. Furthermore, it was suspected that a further contribution to the improvement of the leaching potential could be obtained by the detachment of unbound fine particles weakly adhering to coarser ash fractions, and therefore not screened in standard screening operations in BA processing plants.

The greyish coating layer observed for wet-quenched BA after ageing was removed, displaying the original colors of melt products and inert particles. Specifically, melt products were turned to their original dark brown color and in several cases, their vesicular structure was revealed. It was presumable that repeated collisions cause particle vibrations, in turn capable of detaching mineral phases contained in the ash pores, which are later removed from the process by the continuous turning of the particles. At the same time, refractory materials such as glass, ceramics, and the few metal particles still embedded in the mineral fraction were polished and freed from secondary weathering phases. Therefore, the reduction of salts and PTEs was finally achieved by the sieving out of the progressively generated products of abrasion from the treated coarser particles.

**Figure 5:**
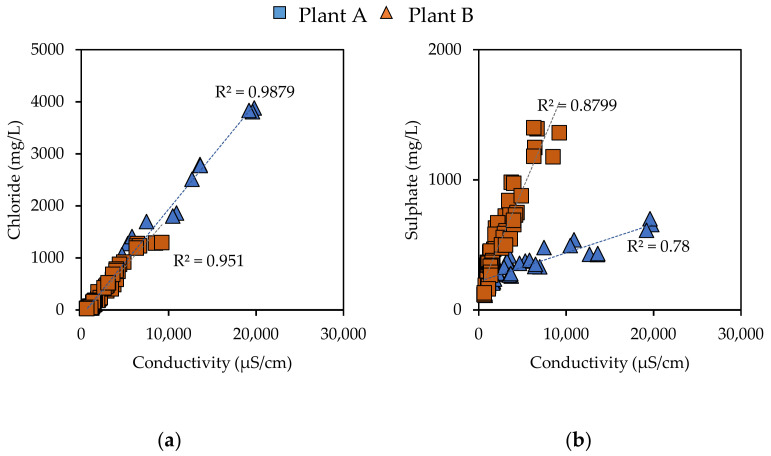
Correlations between electrical conductivity and (**a**) chloride and (**b**) sulphate concentrations in the leachates.

From a qualitative point of view, the removal of the coating layer from BA particles was echoed in an important change in their physical–chemical properties. In all cases, the abraded samples showed a significant decrease in the EC of the leachates (Table 3), which improved with growing abrasion times. Plant A leachates at L/S equal to 0.3 L/kg were characterized by EC values at around 20 mS/cm; the EC value halved at L/S ratio equal to 1 L/kg, and decreased to values below 2 mS/cm for L/S ratios over 3 L/kg. Regarding 60 min and 120 min abrasion times, it was possible to observe a reduction of 30% and 66% on the initial EC, decreased down to values below 1 mS/cm for L/S ratio equal to 4 L/kg. Similar results were achieved for the leachates of the samples from Plant B; the EC of the untreated samples was lower compared to that of the samples from Plant A (7 mS/cm), with the EC value almost halved after 120 min of abrasion time. Even in this case, the final EC values were below 1 mS/cm for L/S equal to 4 L/kg. The analysis of the EC trends clearly demonstrated a significant depletion of the soluble species in the treated BA samples. Linear correlations between EC values and the concentrations of the main salts in the leachates were proven (Figure 5). This, in turn, could be directly correlated with a significant reduction of chloride (r (58) = 0.98, *p* < 0.01) and sulphate (r (58) = 0.94, *p* < 0.01) concentrations. Thus, the EC could be used as a quick in-field control parameter for the evaluation of the effectiveness of an abrasion process.

**Figure 6:**
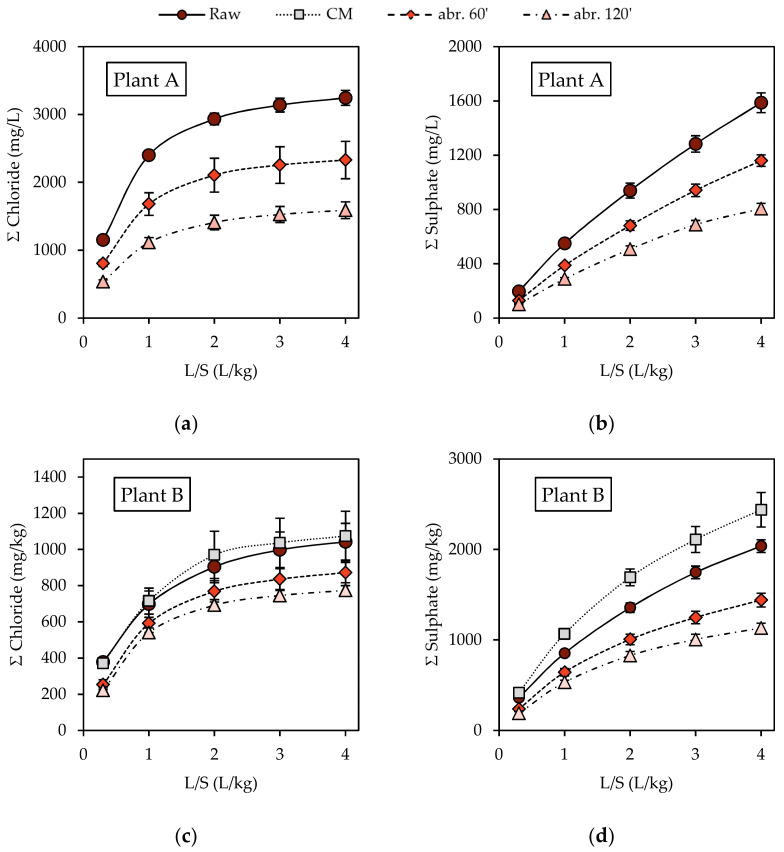
Trends of (**a**) chlorides and (**b**) sulphates’ cumulative release from the raw BA samples, and of (**c**) chlorides and (**d**) sulphates’ cumulative release after the abrasion tests (raw: untreated samples; CM: samples treated in the concrete mixer for 240 min; abr. 60 and abr. 120: samples treated in the sieving drum unit for 60 min and 120 min).

The cumulative releases of chloride and sulphate ions in the leachates before and after the abrasion tests are shown in Figure 6 and in Table 4. Chloride salts, which are highly soluble, exhibited leaching profiles characterized by a relevant drop in concentrations that are already at low L/S ratios. With regard to the samples from Plant A, the unprocessed BA presented initial chloride concentrations close to 3800 mg/L at an L/S ratio of 0.3 L/kg (Figure 6a). These values were halved at an L/S ratio equal to 1 due to the high solubility of chlorides. Between L/S ratios equal to 1 and 2 L/kg, it was possible to observe the shift from the diffusion-controlled dissolution to the reaction-controlled dissolution of low-solubility mineral chloride phases. This was clearly visible in the cumulative concentration curve of chlorides, which tends to flatten for L/S ratios > 2 L/kg. Regarding the samples from Plant B, the processed samples showed decreasing initial chloride concentrations for growing abrasion times (Figure 6c). The effect of the abrasion was reflected mostly at low L/S ratios, where concentrations of 2700 mg Cl^−^/L and 1800 mg Cl^−^/L were observed at an L/S ratio of 0.3 L/kg, for 60 min and 120 min of abrasion time, respectively. This results in the flattening of the cumulative release profile at an L/S ratio equal to 1 L/kg, and the cumulative release limited to 2300 mg/kg and 1600 mg/kg for 60 min and 120 min of abrasion time, respectively. Similar behaviour was observed for the samples from Plant B. In this case, the unprocessed ash and the samples abraded in the concrete mixer showed comparable results. This could be attributed to the establishment of adhesive electrostatic forces between the fine material not promptly removed from the processing unit and the coarse aggregates. However, the concrete mixer abrasion unit was not further investigated. On the other hand, the cumulative chloride release at an L/S equal to 4 L/kg showed a reduction in the total released chlorides for the treated samples. This resulted in a depletion from circa 1050 mg Cl^−^/kg for raw BA from Plant B, to 870 and 770 mg Cl^−^/kg after 60 min and 120 min of abrasion time, respectively.

The sulphates’ leaching trends (Figure 6) were slightly different from those of chlorides, exhibiting a slower concentration decay and resulting in an almost linear cumulative release profile. This could be explained by the sparing solubility of sulphate species such as ettringite, which are governed by reaction-controlled dissolution. Hence, considering the samples collected from Plant A, concentrations of sulphates in the leachate derived from the raw samples (Figure 6b) decreased from 650 mg SO_4_^2−^/L at L/S equal to 0.3 L/kg, down to approximately 300 mg SO_4_^2−^/L at L/S equal to 4 L/kg. However, with regard to the samples from Plant B, the untreated ones were characterized by higher concentrations of sulphate in leachates (Figure 6d); more specifically, it was possible to observe a leaching profile that shared the features of both the reaction-controlled and diffusion-controlled dissolution of salts. Significant reductions in the concentration of sulphates were observed between L/S 0.3 and 1 L/kg, probably due to the presence of large amount of sulphate salts. Their depletion occurred especially until L/S equal to 1, after which a constant decrease was observed (Figure 6d). For the treated samples, a reduction in sulphate concentrations with growing abrasion times was observed in all samples. In contrast to the case of chlorides, the flattening of the cumulative release profiles for sulphate was partially appreciable after 120 min of abrasion time for L/S = 4 L/kg. Regarding the samples from Plant A, the cumulative release decreased from 1600 mg SO_4_^2−^/L down to around 1150 mg SO_4_^2−^/L (after 60 min abrasion) and 800 mg SO_4_^2−^/L (after 20 min abrasion). For samples derived from Plant B, the cumulative release of untreated material was over 2000 mg SO_4_^2−^/L and reduced to 1450 mg SO_4_^2−^/L and 1150 mg SO_4_^2−^/L after 60 min and 120 min of abrasion times, respectively.

#### 3.2.2. Release of Other Compounds

The values for the cumulative release of major and minor elements in the leachates are displayed in Table 4. Chloride and sulphate trends have been previously discussed. The superficial abrasion was demonstrated to be capable of reducing DOC cumulative release to 39%, which is, together with sulphate, the main reason for copper release, as suggested by several studies [36,37] and also confirmed by this work. Despite the high standard deviations obtained by measuring Cu concentrations in the leachates, the decrease of DOC could be the cause of a drastic reduction in Cu concentrations. Furthermore, a reduction of both Cu and DOC after the treatment with the concrete mixer was observed (data not shown). This trend was not observed for other elements, suggesting an auxiliary mechanism acting during the abrasion. However, the reduction was expected to be less effective with the extension of the process duration. As mentioned in the introduction, salts and PTEs during ageing acted as a coating layer on BA particles. By extending the abrasion time enough to completely wear out the outer particle shell, surfaces of minerals formed within the incineration chamber and refractory materials started to be abraded. This behavior explained the higher measure of iron and nickel concentrations in the leachates after 120 min of abrasion time (Table 4). Iron in BA is in the form of non-soluble minerals, and despite its concentration in BA (31–150 g Fe/kg BA) (Astrup et al., 2016), its concentrations in the leachates were relatively low. Hence, what could be hypothesized is that by prolonging the abrasion time, iron minerals start to wear out, leading to increasing iron concentrations in the leachates. As for the main soluble species, the release of specific PTEs as Cu, Cr, and Mo can be tracked by following the progressive washout of the main salts. Molybdenum present as oxyanion MoO_4_^2−^ is reported to exhibit high solubility, although the formation of minerals such as powellite (CaMoO_4_) might have an influence on the leaching behavior [38]. However, in this study, notwithstanding a significant relationship with sulphate concentrations, Mo showed a slightly higher correlation with chloride concentrations (r (58) = 0.97, *p* < 0.001).

Another important piece of information obtained from exploring the correlations among the different elements is the speciation of the soluble salts. The main chloride forms in BA are halite (NaCl) and sylvite (KCl) [18]. This was also proven in this work, where the chloride correlations with K and Na concentrations in the leachates (detailed in the Supplementary Materials) suggested that halite was the primary cause of chloride release (Cl–Na: r (58) = 0.99, *p* < 0.001; Cl–K: r (58) = 0.99, *p* < 0.001). Similarly, the correlation between calcium and sulphate concentrations (r (58) = 0.98, *p* < 0.001) suggested the dissolution of gypsum, a typical product resulting from BA weathering processes [16].

**Figure 7:**
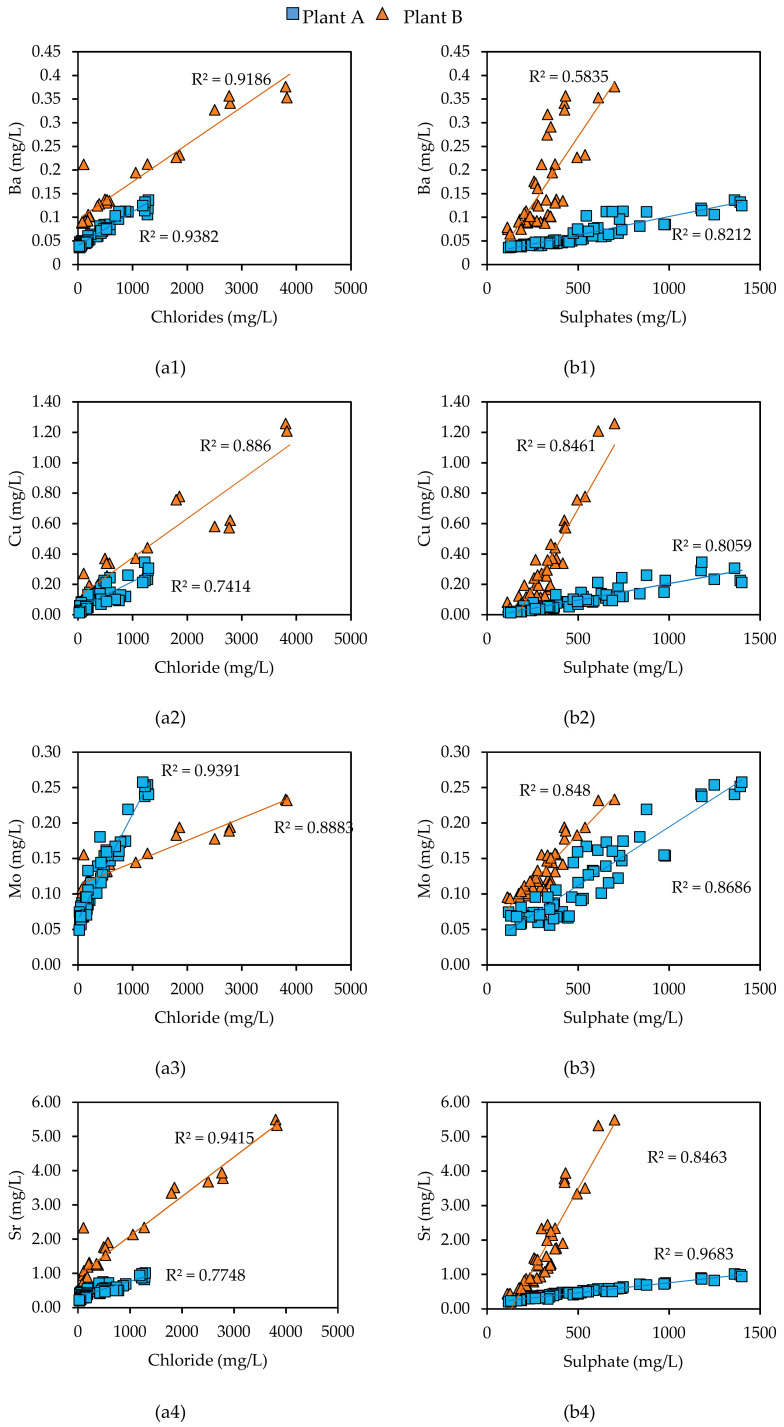
Correlations between chlorides (**a1**–**a4**) and sulphates (**b1**–**b4**) and Ba, Cu, Mo, and Sr in the leachates derived from the column leaching tests.

The efficiency of the proposed dry treatment was finally assessed by studying the correlations between the major elements and the PTEs. The statistical analysis performed on all the concentration values measured in the leachates (Tables S1 and S2, Supplementary Materials) allowed us to obtain some interesting results (Figure 7). Chloride and sulphate concentrations in the leachates showed a good correlation with Ba, Cu, Mo, and Sr. It can be concluded that the dissolution trends of the PTEs were coherent with the behavior followed by the bearing salts (e.g., mainly chlorides and sulphates), present in the outer layers coating the BA particles.

The variables portrayed in Figure 7 show a large positive relationship; the values of the coefficient R^2^ determine the quality of the fitting of the linear correlation model applied to the considered variables. Nonetheless, the strength of the correlation between the variables was expressed by the Pearson coefficient R (see Supplementary Materials Tables S1 and S2). All the portrayed relationships displayed a significance level below the threshold α ≤ 0.05.

## 4. Conclusions

This work investigated at the laboratory scale a dry treatment process based on abrasion, used for the removal of salts and PTEs from BA mineral fraction particles. Two different abrasion units were developed and operated at different times. The best results were obtained using an attrition unit equipped with a screen for the removal of the progressively generated fines. In this case, a relevant drop in the electrical conductivity was associated to a reduction of chlorides (up to 26%) and sulphates (up to 44%), in turn coherent with lower amounts of PTEs released from BA in percolation tests (up to 53% for chromium, 60% for copper, and 8% for molybdenum). On the other hand, prolonged abrasion times showed slightly higher concentrations of Fe, Co, and Ni in leachates, suggesting that the process reached deeper layers of iron-containing incineration products. Further tests are required to assess the release of these metals in aggregate mixtures and its correlation with the duration of abrasion. The statistical analysis revealed good correlations between chloride and sulphate concentrations in the leachates, and the concentrations of barium, copper, molybdenum, and strontium, thus revealing consistent behavior for the major salts and the cited minor components present in the layer surrounding the BA particles. Overall, the studied approach is a valid alternative to wet processes for the reduction of the leaching potential of salts and PTEs in coarse bottom ash samples. However, two key issues need to be further investigated: the specific effect of abrasion on BA components (inerts, refractory materials, and incineration products), and the influence of abrasion on the release of PTEs in the leachates.

The results of our research cannot be compared with previous studies on the topic due to the originality of the process, making it impossible to contextualize our findings. As a general conclusion, any BA valorization strategy should consider that the parent material could affect the overall impact of the results of the abrasion because of two key issues. Firstly, the chemical composition: salts and PTEs, if included in BA composition (e.g., sulphates in residues from renovation activities and/or gypsum), would be released as fine particles. Therefore, it was necessary to equip the abrasion unit with a screen for the removal of fines to limit their recirculation and adhesion to the abraded particles. Secondly, the material hardness: the lower it was, the more effective the abrasion was. Refractory materials (glass, ceramics) and incineration products (silicates/pyroxenes, oxides/spinels, and hematite) commonly found in BA are harder than the weathering phases, making possible their further removal through abrasion. In the absence of these harder fractions, it was predictable that salts and PTEs would land directly with the fines that had already been separated in the BA processing plants.

## Figures and Tables

**Table 1:** Empirical description of the grinding actions at different percentages of C_s_. Numbers from 1 to 3 indicate the ascending degree of action (adapted from Gupta and Yan 2016 [30]).

% C_s_	Sliding	Cascading	Centrifuging
10%	3	-	-
20%	3	-	-
30%	3	1	-
40%	2	1	-
50%	2	1	1
60%	2	2	1
70%	1	3	3
80%	1	3	2
90%	–	2	3

**Table 2:** Parameters of the abrasion tests performed on the samples collected at plants A and B.

Sample ID	Processing Unit	Duration (min)	Plant A (2–40 mm)	Plant B (4–26 mm)
Raw	Untreated sample	-	✓	✓
CM	Concrete Mixer	240	✗	✓
Abr. 60	Sieving drum	60	✓	✓
Abr. 120	Sieving drum	120	✓	✓

**Table 3:** Electrical conductivity values measured in leachates, obtained at different L/S ratios. (Raw: untreated sample; 240 CM: samples treated in the concrete mixer for 240 min; 60–120 Abr.: samples treated in the sieving drum for 60 and 120 min abrasion (Abr.) time respectively).

**L/S** **(L/kg)**	**Plant A**			
	**Raw**	**240 CM**	**Abr. 60**	**Abr. 120**
	**(mS/cm)**			
0.3	19.53 ± 0.31	Not investigated	13.31 ± 0.54	6.64 ± 0.30
1.0	9.63 ± 1.87		5.27 ± 0.57	3.64 ± 0.12
2.0	3.35 ± 0.13		2.47 ± 0.37	1.72 ± 0.10
3.0	2.03 ± 0.07		1.38 ± 0.10	1.03 ± 0.03
4.0	1.53 ± 0.07		0.93 ± 0.03	0.73 ± 0.01
**L/S** **(L/kg)**	**Plant B**			
	**Raw**	**240 CM**	**Abr. 60**	**Abr. 120**
	**(mS/cm)**			
0.3	7.08 ± 1.24	7.40 ± 1.59	4.50 ± 0.35	3.88 ± 0.22
1.0	3.19 ± 0.35	3.73 ± 0.29	2.97 ± 0.16	2.77 ± 0.27
2.0	1.85 ± 0.12	2.00 ± 0.17	1.56 ± 0.07	1.37 ± 0.04
3.0	1.35 ± 0.08	1.24 ± 0.12	1.02 ± 0.02	1.10 ± 0.04
4.0	1.18 ± 0.06	1.07 ± 0.12	0.86 ± 0.19	0.63 ± 0.03

**Table 4:** Cumulative release for the elements considered at an L/S equal to 4 L/kg. (The results obtained from the concrete mixer are not shown). Reduction rates are calculated on the raw sample for increasing abrasion (Abr.) times.

Parameter	Plant A					Plant B				
	Concentration			Reduction Rate		Concentration			Reduction Rate	
	Raw	Abr. 60′	Abr. 120′	Abr. 60′	Abr. 120′	Raw	Abr. 60′	Abr. 120′	Abr. 60′	Abr. 120′
**Major Elements (mg/kg)**										
**Cl^−^**	3244 ± 111	2329 ± 275	1589 ± 123	−28%	−51%	1043 ± 102	872 ± 56	774 ± 27	−16%	−26%
**SO_4_^2−^**	1587 ± 73	1160 ± 42	806 ± 39	−27%	−49%	2037 ± 70	1441 ± 76	1132 ± 54	−29%	−44%
**DOC**	264 ± 16	180 ± 11	120 ± 9	−32%	−55%	157 ± 24	117 ± 3	96 ± 5	−26%	−39%
**Ca**	1360 ± 134	887 ± 33	668 ± 23	−35%	−51%	529 ± 29	404 ± 16	325 ± 8	−24%	−39%
**K**	627 ± 19	454 ± 36	345 ± 20	−27%	−45%	249 ± 21	203 ± 11	176 ± 7	−19%	−29%
**Na**	1224 ± 33	912 ± 78	711 ± 46	−25%	−42%	834 ± 80	674 ± 45	587 ± 33	−19%	−30%
**Minor Elements (μg/kg)**										
**Al**	3913 ± 769	9822 ± 1267	17290 ± 4085	151%	342%	7222 ± 521	6192 ± 167	5964 ± 195	−14%	−17%
**B**	495 ± 126	373 ± 13	698 ± 167	−25%	41%	1891 ± 124	1593 ± 51	1815 ± 228	−16%	−4%
**Ba**	639 ± 62	557 ± 15	476 ± 28	−13%	−25%	220 ± 9	219 ± 12	206 ± 5	−1%	−7%
**Co**	11 ± 4	16 ± 2	18 ± 3	44%	55%	37 ± 4	49 ± 11	57 ± 3	33%	52%
**Cr**	276 ± 45	152 ± 17	141 ± 15	−45%	−49%	469 ± 76	290 ± 67	221 ± 43	−38%	−53%
**Cu**	1613 ± 76	868 ± 61	619 ± 135	−46%	−62%	448 ± 118	301 ± 172	181 ± 31	−33%	−60%
**Fe**	181 ± 65	206 ± 41	216 ± 42	14%	19%	323 ± 83	245 ± 19	316 ± 49	−24%	−2%
**Li**	500 ± 42	285 ± 17	184 ± 4	−43%	−63%	94 ± 8	56 ± 6	40 ± 6	−41%	−58%
**Mg**	351 ± 54	337 ± 8	460 ± 44	−4%	31%	347 ± 36	284 ± 53	337 ± 21	−18%	−3%
**Mn**	9 ± 1	13 ± 2	10 ± 2	40%	6%	20 ± 3	18 ± 3	22 ± 2	−10%	10%
**Mo**	586 ± 14	498 ± 19	440 ± 14	−15%	−25%	400 ± 18	375 ± 46	368 ± 39	−6%	−8%
**Ni**	38 ± 16	27 ± 17	30 ± 4	−28%	−21%	67 ± 14	96 ± 17	104 ± 15	43%	54%
**Sr**	8528 ± 700	5552 ± 263	3589 ± 39	−35%	−58%	1955 ± 89	1555 ± 118	1208 ± 38	−20%	−38%
**Ti**	45 ± 1	43 ± 3	43 ± 1	−5%	−6%	99 ± 1	100 ± 3	100 ± 1	1%	0%
**V**	203 ± 19	144 ± 6	126 ± 9	−29%	−38%	145 ± 8	144 ± 4	143 ± 5	0%	−1%
**Zn**	166 ± 37	143 ± 65	110 ± 33	−14%	−34%	80 ± 31	265 ± 138	20 ± 1	233%	−75%

## Data Availability

The full data related to this research are not publicly available due to the confidentiality agreement signed by the Consortium of BASH-TREAT project.

## Supplementary Materials

The following are available online at https://www.mdpi.com/article/10.3390/ma14113133/s1, Table S1: Results of statistical analysis of the correlations among the major and minor compounds analysed in the leachates (samples of bottom ash mineral fraction from PLANT A); Table S2: Results of statistical analysis of the correlations among the major and minor compounds analysed in the leachates (samples of bottom ash mineral fraction from PLANT B).
