# Supplementary material for: A Novel Dry Treatment for Municipal Solid Waste Incineration Bottom Ash for the Reduction of Salts and Potential Toxic Elements
Source: Materials (Basel). 2021 Jun 7;14(11):3133. doi: 10.3390/ma14113133 (PMC8201139; doi:10.3390/ma14113133)
Supplement: Supplementary file 1 [file materials-14-03133-s001.zip › materials-1203344-supplementary.pdf]

materials

Supplementary Material

A Novel Dry Treatment for Municipal Solid Waste Incineration Bottom Ash for the Reduction of Salts and Potential Toxic Elements

Marco Abis [1,*], Martina Bruno [2], Franz-Georg Simon [3], Raul Grönholm [4], Michel Hoppe [5], Kerstin Kuchta [1] and Silvia Fiore [2,*]

1 SRWM (Sustainable Resource and Waste Management), Hamburg University of Technology, 21079 Hamburg, Germany; kuchta@tuhh.de
2 DIATI (Department of Engineering for Environment, Land and Infrastructures), Politecnico di Torino, 10129 Torino, Italy; martina.bruno@polito.it
3 Bundesanstalt für Materialforschung und −Prüfung (BAM), 12200 Berlin, Germany; franz−georg.simon@bam.de
4 Sysav Utveckling AB, 20025 Malmö, Sweden; Raul.Gronholm@sysav.se
5 Heidemann Recycling GmbH, 28277 Bremen, Germany; m.hoppe@heidemann−recycling.de
* Correspondence: marco.abis@tuhh.de (M.A.); silvia.fiore@polito.it (S.F.)

Citation: Abis, M.; Bruno, M.; Simon, F.−G.; Grönholm, R.; Hoppe, M.; Kuchta, K.; Fiore, S. A Novel Dry Treatment for Municipal Solid Waste Incineration Bottom Ash for the Reduction of Salts and Potential Toxic Elements. *Materials* **2021**, *14*, 3133. https://doi.org/10.3390/ma14113133

Publisher's Note: MDPI stays neutral with regard to jurisdictional claims in published maps and institutional affiliations.

Copyright: © 2021 by the authors. Licensee MDPI, Basel, Switzerland. This article is an open access article distributed under the terms and conditions of the Creative Commons Attribution (CC BY) license (http://creativecommons.org/licenses/by/4.0/).

Table SI. Results of statistical analysis of the correlations among the major and minor compounds analysed in the leachates (samples of bottom ash mineral fraction from PLANT A).

Data sets	Pearson Correlation	Significance (Two Tailed)	95% Confidence Interval (Two Tailed)	
			Upper	Lower
Chlorides–Sulphates	0.852	0.000	0.745	0.916
Chlorides–Al	0.310	0.046	0.007	0.561
Chlorides–B	−0.369	0.016	−0.605	−0.074
Chlorides–Ba	0.950	0.000	0.908	0.973
Chlorides–Be	0.280	0.073	−0.026	0.538
Chlorides–Ca	0.913	0.000	0.842	0.952
Chlorides–Co	0.350	0.023	0.052	0.591
Chlorides–Cr	0.714	0.000	0.523	0.836
Chlorides–Cu	0.925	0.000	0.864	0.959
Chlorides–Fe	0.482	0.001	0.208	0.685
Chlorides–K	0.996	0.000	0.993	0.998
Chlorides–Li	0.928	0.000	0.870	0.961
Chlorides–Mg	0.630	0.000	0.403	0.784
Chlorides–Mn	0.522	0.000	0.259	0.713
Chlorides–Mo	0.922	0.000	0.859	0.958
Chlorides–Na	0.994	0.000	0.990	0.997
Chlorides–Ni				
Chlorides–Sr	0.952	0.000	0.912	0.974
Chlorides–Ti				
Chlorides–V	0.792	0.000	0.643	0.883
Chlorides–Zn	0.247	0.114	−0.061	0.513
Sulphates–Al	−0.104	0.511	−0.396	0.206
Sulphates–B	−0.457	0.002	−0.668	−0.178
Sulphates–Ba	0.762	0.000	0.596	0.866
Sulphates–Be	0.177	0.262	−0.134	0.456
Sulphates–Ca	0.931	0.000	0.874	0.963
Sulphates–Co	0.003	0.987	−0.302	0.306
Sulphates–Cr	0.822	0.000	0.690	0.901
Sulphates–Cu	0.920	0.000	0.856	0.957
Sulphates–Fe	0.193	0.220	−0.118	0.470
Sulphates–K	0.808	0.000	0.668	0.893
Sulphates–Li	0.935	0.000	0.881	0.965
Sulphates–Mg	0.379	0.013	0.084	0.612
Sulphates–Mn	0.225	0.151	−0.084	0.495
Sulphates–Mo	0.907	0.000	0.833	0.949
Sulphates–Na	0.800	0.000	0.655	0.888
Sulphates–Ni				
Sulphates–Sr	0.920	0.000	0.855	0.956
Sulphates–Ti				
Sulphates–V	0.861	0.000	0.754	0.923
Sulphates–Zn	0.268	0.087	−0.040	0.528
Al–B	0.234	0.136	−0.075	0.502
Al–Ba	0.436	0.004	0.153	0.654
Al–Be	0.225	0.153	−0.085	0.495
Al–Ca	0.094	0.555	−0.216	0.387
Al–Co	0.436	0.004	0.152	0.653

Al–Cr	−0.172	0.275	−0.453	0.139
Al–Cu	0.023	0.883	−0.282	0.325
Al–Fe	0.640	0.000	0.417	0.790
Al–K	0.363	0.018	0.066	0.601
Al–Li	0.063	0.693	−0.246	0.360
Al–Mg	0.515	0.000	0.250	0.708
Al–Mn	0.641	0.000	0.419	0.791
Al–Mo	0.120	0.449	−0.191	0.409
Al–Na	0.383	0.012	0.090	0.615
Al–Ni				
Al–Sr	0.109	0.494	−0.202	0.399
Al–Ti				
Al–V	−0.100	0.530	−0.392	0.211
Al–Zn	0.075	0.636	−0.234	0.371
B–Ba	−0.286	0.066	−0.543	0.020
B–Be	−0.125	0.431	−0.413	0.186
B–Ca	−0.304	0.050	−0.557	−0.001
B–Co	0.160	0.311	−0.151	0.443
B–Cr	−0.107	0.500	−0.398	0.203
B–Cu	−0.383	0.012	−0.615	−0.089
B–Fe	−0.093	0.556	−0.386	0.217
B–K	−0.354	0.022	−0.594	−0.056
B–Li	−0.384	0.012	−0.616	−0.090
B–Mg	0.111	0.486	−0.200	0.401
B–Mn	−0.110	0.488	−0.401	0.201
B–Mo	−0.357	0.020	−0.596	−0.060
B–Na	−0.356	0.021	−0.595	−0.058
B–Ni				
B–Sr	−0.384	0.012	−0.616	−0.091
B–Ti				
B–V	−0.215	0.172	−0.487	0.095
B–Zn	0.002	0.989	−0.302	0.306
Ba–Be	0.274	0.079	−0.032	0.534
Ba–Ca	0.903	0.000	0.826	0.947
Ba–Co	0.364	0.018	0.068	0.602
Ba–Cr	0.661	0.000	0.447	0.804
Ba–Cu	0.844	0.000	0.726	0.914
Ba–Fe	0.615	0.000	0.383	0.774
Ba–K	0.969	0.000	0.943	0.983
Ba–Li	0.890	0.000	0.804	0.940
Ba–Mg	0.679	0.000	0.473	0.815
Ba–Mn	0.635	0.000	0.411	0.787
Ba–Mo	0.903	0.000	0.826	0.947
Ba–Na	0.970	0.000	0.945	0.984
Ba–Ni				
Ba–Sr	0.922	0.000	0.859	0.958
Ba–Ti				
Ba–V	0.780	0.000	0.624	0.876
Ba–Zn	0.276	0.077	−0.030	0.535
Be–Ca	0.226	0.150	−0.084	0.496
Be–Co	−0.069	0.664	−0.365	0.240

Be–Cr	0.092	0.562	−0.218	0.385
Be–Cu	0.244	0.120	−0.065	0.510
Be–Fe	0.079	0.617	−0.230	0.374
Be–K	0.279	0.074	−0.027	0.537
Be–Li	0.237	0.131	−0.072	0.504
Be–Mg	−0.013	0.933	−0.316	0.292
Be–Mn	0.054	0.736	−0.254	0.352
Be–Mo	0.246	0.117	−0.063	0.511
Be–Na	0.285	0.067	−0.020	0.542
Be–Ni				
Be–Sr	0.238	0.128	−0.071	0.506
Be–Ti				
Be–V	0.100	0.527	−0.210	0.392
Be–Zn	−0.173	0.272	−0.453	0.138
Ca–Co	0.145	0.359	−0.166	0.430
Ca–Cr	0.882	0.000	0.789	0.935
Ca–Cu	0.957	0.000	0.921	0.977
Ca–Fe	0.316	0.041	0.014	0.566
Ca–K	0.910	0.000	0.839	0.951
Ca–Li	0.987	0.000	0.975	0.993
Ca–Mg	0.500	0.001	0.231	0.698
Ca–Mn	0.353	0.022	0.055	0.593
Ca–Mo	0.980	0.000	0.962	0.989
Ca–Na	0.904	0.000	0.828	0.948
Ca–Ni				
Ca–Sr	0.987	0.000	0.976	0.993
Ca–Ti				
Ca–V	0.931	0.000	0.875	0.963
Ca–Zn	0.286	0.066	−0.020	0.543
Co–Cr	−0.021	0.893	−0.323	0.284
Co–Cu	0.140	0.377	−0.171	0.426
Co–Fe	0.551	0.000	0.297	0.732
Co–K	0.359	0.020	0.062	0.598
Co–Li	0.126	0.426	−0.185	0.414
Co–Mg	0.656	0.000	0.440	0.800
Co–Mn	0.572	0.000	0.325	0.746
Co–Mo	0.177	0.263	−0.134	0.456
Co–Na	0.361	0.019	0.065	0.599
Co–Ni				
Co–Sr	0.189	0.232	−0.122	0.466
Co–Ti				
Co–V	0.153	0.333	−0.158	0.437
Co–Zn	0.149	0.347	−0.163	0.433
Cr–Cu	0.881	0.000	0.787	0.934
Cr–Fe	−0.014	0.929	−0.317	0.291
Cr–K	0.696	0.000	0.497	0.825
Cr–Li	0.863	0.000	0.758	0.924
Cr–Mg	0.327	0.034	0.026	0.574
Cr–Mn	0.013	0.937	−0.292	0.315
Cr–Mo	0.836	0.000	0.713	0.909
Cr–Na	0.681	0.000	0.475	0.816

Cr–Ni				
Cr–Sr	0.838	0.000	0.717	0.910
Cr–Ti				
Cr–V	0.882	0.000	0.791	0.935
Cr–Zn	0.239	0.127	−0.070	0.506
Cu–Fe	0.190	0.229	−0.121	0.467
Cu–K	0.910	0.000	0.837	0.951
Cu–Li	0.978	0.000	0.959	0.988
Cu–Mg	0.425	0.005	0.139	0.645
Cu–Mn	0.230	0.143	−0.079	0.499
Cu–Mo	0.950	0.000	0.908	0.973
Cu–Na	0.902	0.000	0.825	0.947
Cu–Ni				
Cu–Sr	0.966	0.000	0.937	0.982
Cu–Ti				
Cu–V	0.891	0.000	0.805	0.940
Cu–Zn	0.213	0.175	−0.097	0.486
Fe–K	0.519	0.000	0.256	0.711
Fe–Li	0.274	0.079	−0.033	0.534
Fe–Mg	0.691	0.000	0.490	0.822
Fe–Mn	0.863	0.000	0.758	0.924
Fe–Mo	0.345	0.025	0.045	0.587
Fe–Na	0.530	0.000	0.269	0.718
Fe–Ni				
Fe–Sr	0.351	0.023	0.053	0.592
Fe–Ti				
Fe–V	0.185	0.241	−0.126	0.463
Fe–Zn	0.238	0.129	−0.071	0.505
K–Li	0.923	0.000	0.861	0.958
K–Mg	0.642	0.000	0.420	0.791
K–Mn	0.553	0.000	0.299	0.733
K–Mo	0.918	0.000	0.852	0.955
K–Na	0.999	0.000	0.999	1.000
K–Ni				
K–Sr	0.947	0.000	0.903	0.971
K–Ti				
K–V	0.786	0.000	0.633	0.880
K–Zn	0.258	0.099	−0.050	0.521
Li–Mg	0.444	0.003	0.162	0.659
Li–Mn	0.312	0.044	0.009	0.563
Li–Mo	0.974	0.000	0.951	0.986
Li–Na	0.917	0.000	0.850	0.955
Li–Ni				
Li–Sr	0.992	0.000	0.984	0.996
Li–Ti				
Li–V	0.916	0.000	0.849	0.954
Li–Zn	0.262	0.094	−0.046	0.524
Mg–Mn	0.728	0.000	0.545	0.845
Mg–Mo	0.493	0.001	0.222	0.693
Mg–Na	0.638	0.000	0.415	0.789
Mg–Ni				

Mg–Sr	0.515	0.000	0.250	0.708
Mg–Ti				
Mg–V	0.405	0.008	0.115	0.631
Mg–Zn	0.267	0.087	−0.040	0.528
Mn–Mo	0.381	0.013	0.087	0.614
Mn–Na	0.567	0.000	0.318	0.743
Mn–Ni				
Mn–Sr	0.391	0.010	0.099	0.621
Mn–Ti				
Mn–V	0.194	0.219	−0.117	0.470
Mn–Zn	0.242	0.123	−0.067	0.508
Mo–Na	0.914	0.000	0.845	0.953
Mo–Ni				
Mo–Sr	0.979	0.000	0.962	0.989
Mo–Ti				
Mo–V	0.898	0.000	0.817	0.944
Mo–Zn	0.267	0.088	−0.040	0.528
Na–Ni				
Na–Sr	0.941	0.000	0.893	0.968
Na–Ti				
Na–V	0.773	0.000	0.613	0.872
Na–Zn	0.253	0.105	−0.055	0.518
Ni–Sr				
Ni–Ti				
Ni–V				
Ni–Zn				
Sr–Ti				
Sr–V	0.910	0.000	0.838	0.951
Sr–Zn	0.275	0.078	−0.032	0.534
Ti–V				
Ti–Zn				
V–Zn	0.231	0.141	−0.079	0.500

Table SII. Results of statistical analysis of the correlations among the major and minor compounds analysed in the leachates (samples of bottom ash mineral fraction from PLANT B).

Data sets	**Pearson Correlation**	**Significance (Two Tailed)**	**95% Confidence Interval (Two Tailed)**	
			**Lower**	**Upper**
Chlorides–Sulphates	0.922	0.000	0.871	0.953
Chlorides–TOC	0.975	0.000	0.959	0.985
Chlorides–Al	−0.585	0.000	−0.732	−0.387
Chlorides–B	0.707	0.000	0.551	0.815
Chlorides–Ba	0.970	0.000	0.950	0.982
Chlorides–Be	0.191	0.147	−0.068	0.426
Chlorides–Ca	0.842	0.000	0.746	0.903
Chlorides–Co	−0.120	0.366	−0.365	0.141
Chlorides–Cr	0.783	0.000	0.659	0.866
Chlorides–Cu	0.853	0.000	0.764	0.910
Chlorides–Fe	0.359	0.005	0.113	0.563
Chlorides–K	0.993	0.000	0.988	0.996
Chlorides–Li	0.701	0.000	0.543	0.812
Chlorides–Mg	0.807	0.000	0.695	0.881
Chlorides–Mn	0.632	0.000	0.449	0.764
Chlorides–Mo	0.968	0.000	0.946	0.981
Chlorides–Na	0.995	0.000	0.991	0.997
Chlorides–Ni	−0.019	0.884	−0.274	0.238
Chlorides–Sr	0.879	0.000	0.804	0.927
Chlorides–Ti	−0.012	0.927	−0.267	0.245
Chlorides–V	0.234	0.074	−0.023	0.463
Chlorides–Zn	0.177	0.179	−0.082	0.415
Sulphates–TOC	0.931	0.000	0.886	0.959
Sulphates–Al	−0.511	0.000	−0.679	−0.294
Sulphates–B	0.562	0.000	0.357	0.715
Sulphates–Ba	0.906	0.000	0.846	0.943
Sulphates–Be	0.192	0.146	−0.068	0.427
Sulphates–Ca	0.976	0.000	0.960	0.986
Sulphates–Co	−0.294	0.024	−0.511	−0.041
Sulphates–Cr	0.716	0.000	0.563	0.822
Sulphates–Cu	0.895	0.000	0.828	0.936
Sulphates–Fe	0.203	0.122	−0.056	0.437
Sulphates–K	0.942	0.000	0.905	0.965
Sulphates–Li	0.872	0.000	0.792	0.922
Sulphates–Mg	0.691	0.000	0.529	0.805
Sulphates–Mn	0.493	0.000	0.272	0.665
Sulphates–Mo	0.928	0.000	0.882	0.957
Sulphates–Na	0.929	0.000	0.884	0.958
Sulphates–Ni	−0.221	0.092	−0.452	0.037
Sulphates–Sr	0.984	0.000	0.973	0.991
Sulphates–Ti	−0.042	0.754	−0.295	0.217
Sulphates–V	0.136	0.305	−0.125	0.379
Sulphates–Zn	0.240	0.067	−0.017	0.468
TOC–Al	−0.484	0.000	−0.658	−0.260
TOC–B	0.679	0.000	0.512	0.797
TOC–Ba	0.923	0.000	0.874	0.954

TOC–Be	0.208	0.113	−0.050	0.441
TOC–Ca	0.860	0.000	0.774	0.914
TOC–Co	−0.187	0.156	−0.423	0.072
TOC–Cr	0.858	0.000	0.772	0.914
TOC–Cu	0.900	0.000	0.836	0.939
TOC–Fe	0.270	0.039	0.015	0.492
TOC–K	0.983	0.000	0.972	0.990
TOC–Li	0.719	0.000	0.567	0.823
TOC–Mg	0.726	0.000	0.577	0.828
TOC–Mn	0.576	0.000	0.375	0.725
TOC–Mo	0.949	0.000	0.916	0.970
TOC–Na	0.980	0.000	0.967	0.988
TOC–Ni	−0.122	0.359	−0.366	0.139
TOC–Sr	0.900	0.000	0.838	0.940
TOC–Ti	−0.066	0.619	−0.317	0.193
TOC–V	0.188	0.153	−0.071	0.424
TOC–Zn	0.177	0.179	−0.083	0.415
Al–B	−0.556	0.000	−0.711	−0.350
Al–Ba	−0.649	0.000	−0.776	−0.471
Al–Be	−0.178	0.178	−0.415	0.082
Al–Ca	−0.440	0.000	−0.625	−0.207
Al–Co	−0.032	0.813	−0.285	0.226
Al–Cr	−0.200	0.128	−0.434	0.059
Al–Cu	−0.355	0.006	−0.560	−0.108
Al–Fe	−0.235	0.073	−0.463	0.022
Al–K	−0.576	0.000	−0.725	−0.375
Al–Li	−0.333	0.010	−0.543	−0.084
Al–Mg	−0.551	0.000	−0.707	−0.343
Al–Mn	−0.399	0.002	−0.594	−0.159
Al–Mo	−0.606	0.000	−0.747	−0.415
Al–Na	−0.589	0.000	−0.734	−0.392
Al–Ni	−0.401	0.002	−0.596	−0.161
Al–Sr	−0.452	0.000	−0.635	−0.222
Al–Ti	−0.100	0.451	−0.347	0.160
Al–V	−0.375	0.003	−0.576	−0.132
Al–Zn	−0.261	0.046	−0.485	−0.005
B–Ba	0.708	0.000	0.553	0.816
B–Be	0.088	0.508	−0.172	0.336
B–Ca	0.449	0.000	0.218	0.632
B–Co	−0.130	0.328	−0.373	0.131
B–Cr	0.683	0.000	0.517	0.799
B–Cu	0.537	0.000	0.326	0.697
B–Fe	0.477	0.000	0.252	0.653
B–K	0.712	0.000	0.558	0.819
B–Li	0.265	0.042	0.010	0.488
B–Mg	0.680	0.000	0.513	0.797
B–Mn	0.519	0.000	0.303	0.684
B–Mo	0.674	0.000	0.505	0.793
B–Na	0.726	0.000	0.578	0.828
B–Ni	0.303	0.020	0.051	0.519
B–Sr	0.526	0.000	0.312	0.689

B–Ti	−0.018	0.891	−0.273	0.239
B–V	0.367	0.004	0.122	0.569
B–Zn	−0.062	0.641	−0.313	0.197
Ba–Be	0.179	0.176	−0.081	0.416
Ba–Ca	0.843	0.000	0.749	0.904
Ba–Co	−0.104	0.435	−0.350	0.157
Ba–Cr	0.669	0.000	0.498	0.790
Ba–Cu	0.797	0.000	0.680	0.875
Ba–Fe	0.435	0.001	0.202	0.622
Ba–K	0.967	0.000	0.944	0.980
Ba–Li	0.721	0.000	0.571	0.825
Ba–Mg	0.856	0.000	0.768	0.912
Ba–Mn	0.660	0.000	0.487	0.784
Ba–Mo	0.947	0.000	0.912	0.968
Ba–Na	0.970	0.000	0.950	0.982
Ba–Ni	0.021	0.874	−0.236	0.276
Ba–Sr	0.872	0.000	0.793	0.922
Ba–Ti	−0.005	0.972	−0.260	0.252
Ba–V	0.208	0.113	−0.051	0.441
Ba–Zn	0.205	0.120	−0.054	0.438
Be–Ca	0.179	0.175	−0.081	0.416
Be–Co	0.165	0.211	−0.095	0.404
Be–Cr	0.188	0.153	−0.071	0.424
Be–Cu	0.243	0.063	−0.014	0.470
Be–Fe	0.189	0.152	−0.071	0.424
Be–K	0.205	0.120	−0.054	0.438
Be–Li	0.214	0.104	−0.045	0.446
Be–Mg	0.197	0.134	−0.062	0.432
Be–Mn	0.191	0.148	−0.069	0.426
Be–Mo	0.238	0.070	−0.019	0.466
Be–Na	0.209	0.112	−0.050	0.442
Be–Ni	−0.095	0.472	−0.343	0.165
Be–Sr	0.194	0.141	−0.066	0.429
Be–Ti	0.014	0.914	−0.243	0.270
Be–V	0.037	0.778	−0.221	0.291
Be–Zn	0.319	0.014	0.068	0.532
Ca–Co	−0.336	0.009	−0.545	−0.087
Ca–Cr	0.625	0.000	0.439	0.759
Ca–Cu	0.867	0.000	0.785	0.919
Ca–Fe	0.129	0.329	−0.131	0.373
Ca–K	0.874	0.000	0.796	0.924
Ca–Li	0.923	0.000	0.874	0.954
Ca–Mg	0.624	0.000	0.438	0.759
Ca–Mn	0.423	0.001	0.187	0.613
Ca–Mo	0.860	0.000	0.774	0.915
Ca–Na	0.854	0.000	0.765	0.911
Ca–Ni	−0.304	0.019	−0.520	−0.052
Ca–Sr	0.989	0.000	0.981	0.993
Ca–Ti	−0.074	0.577	−0.324	0.186
Ca–V	0.077	0.560	−0.182	0.327
Ca–Zn	0.269	0.039	0.014	0.491

Co–Cr	−0.243	0.063	−0.470	0.014
Co–Cu	−0.332	0.010	−0.542	−0.083
Co–Fe	0.196	0.136	−0.063	0.431
Co–K	−0.164	0.214	−0.403	0.096
Co–Li	−0.305	0.019	−0.520	−0.053
Co–Mg	0.145	0.273	−0.115	0.387
Co–Mn	0.266	0.041	0.011	0.489
Co–Mo	−0.177	0.181	−0.414	0.083
Co–Na	−0.143	0.279	−0.385	0.117
Co–Ni	0.203	0.124	−0.056	0.436
Co–Sr	−0.334	0.010	−0.543	−0.085
Co–Ti	0.038	0.773	−0.220	0.292
Co–V	0.053	0.691	−0.206	0.305
Co–Zn	−0.012	0.927	−0.267	0.245
Cr–Cu	0.847	0.000	0.755	0.907
Cr–Fe	0.218	0.097	−0.040	0.449
Cr–K	0.804	0.000	0.690	0.879
Cr–Li	0.459	0.000	0.230	0.640
Cr–Mg	0.547	0.000	0.338	0.704
Cr–Mn	0.470	0.000	0.243	0.648
Cr–Mo	0.759	0.000	0.625	0.850
Cr–Na	0.801	0.000	0.685	0.877
Cr–Ni	−0.141	0.286	−0.383	0.119
Cr–Sr	0.696	0.000	0.536	0.808
Cr–Ti	−0.129	0.331	−0.373	0.132
Cr–V	0.131	0.324	−0.130	0.374
Cr–Zn	−0.005	0.969	−0.261	0.251
Cu–Fe	0.132	0.318	−0.128	0.376
Cu–K	0.884	0.000	0.811	0.929
Cu–Li	0.730	0.000	0.583	0.831
Cu–Mg	0.600	0.000	0.407	0.742
Cu–Mn	0.431	0.001	0.197	0.619
Cu–Mo	0.856	0.000	0.768	0.912
Cu–Na	0.870	0.000	0.789	0.921
Cu–Ni	−0.232	0.078	−0.460	0.026
Cu–Sr	0.898	0.000	0.834	0.938
Cu–Ti	−0.047	0.722	−0.300	0.211
Cu–V	0.110	0.407	−0.150	0.356
Cu–Zn	0.203	0.123	−0.056	0.436
Fe–K	0.328	0.011	0.079	0.539
Fe–Li	0.081	0.543	−0.179	0.330
Fe–Mg	0.715	0.000	0.562	0.821
Fe–Mn	0.694	0.000	0.533	0.807
Fe–Mo	0.324	0.012	0.074	0.535
Fe–Na	0.348	0.007	0.101	0.555
Fe–Ni	0.148	0.263	−0.112	0.389
Fe–Sr	0.159	0.229	−0.101	0.399
Fe–Ti	0.028	0.835	−0.230	0.282
Fe–V	0.014	0.917	−0.243	0.269
Fe–Zn	−0.107	0.420	−0.353	0.153
K–Li	0.737	0.000	0.593	0.835

K–Mg	0.791	0.000	0.671	0.871
K–Mn	0.612	0.000	0.422	0.751
K–Mo	0.972	0.000	0.953	0.983
K–Na	0.999	0.000	0.998	0.999
K–Ni	−0.052	0.694	−0.304	0.207
K–Sr	0.910	0.000	0.852	0.946
K–Ti	−0.031	0.815	−0.285	0.227
K–V	0.223	0.090	−0.035	0.453
K–Zn	0.192	0.146	−0.068	0.427
Li–Mg	0.513	0.000	0.296	0.680
Li–Mn	0.359	0.005	0.113	0.563
Li–Mo	0.775	0.000	0.647	0.860
Li–Na	0.715	0.000	0.562	0.821
Li–Ni	−0.323	0.013	−0.535	−0.073
Li–Sr	0.882	0.000	0.808	0.928
Li–Ti	−0.040	0.763	−0.293	0.218
Li–V	−0.046	0.727	−0.299	0.212
Li–Zn	0.256	0.050	0.000	0.481
Mg–Mn	0.843	0.000	0.748	0.904
Mg–Mo	0.769	0.000	0.638	0.856
Mg–Na	0.801	0.000	0.686	0.877
Mg–Ni	0.088	0.507	−0.172	0.337
Mg–Sr	0.656	0.000	0.481	0.781
Mg–Ti	−0.032	0.812	−0.285	0.226
Mg–V	0.168	0.204	−0.092	0.406
Mg–Zn	0.093	0.485	−0.167	0.341
Mn–Mo	0.611	0.000	0.421	0.750
Mn–Na	0.627	0.000	0.442	0.761
Mn–Ni	0.035	0.794	−0.223	0.288
Mn–Sr	0.458	0.000	0.228	0.639
Mn–Ti	−0.038	0.773	−0.292	0.220
Mn–V	0.096	0.467	−0.164	0.344
Mn–Zn	0.078	0.559	−0.182	0.327
Mo–Na	0.974	0.000	0.956	0.984
Mo–Ni	−0.036	0.788	−0.289	0.222
Mo–Sr	0.889	0.000	0.820	0.933
Mo–Ti	0.004	0.977	−0.252	0.260
Mo–V	0.221	0.093	−0.037	0.451
Mo–Zn	0.201	0.127	−0.058	0.435
Na–Ni	−0.029	0.830	−0.283	0.229
Na–Sr	0.893	0.000	0.826	0.935
Na–Ti	−0.022	0.868	−0.277	0.235
Na–V	0.231	0.078	−0.026	0.460
Na–Zn	0.188	0.153	−0.071	0.424
Ni–Sr	−0.262	0.045	−0.485	−0.006
Ni–Ti	0.098	0.460	−0.162	0.345
Ni–V	0.298	0.022	0.046	0.515
Ni–Zn	0.068	0.609	−0.192	0.318
Sr–Ti	−0.071	0.595	−0.321	0.189
Sr–V	0.117	0.378	−0.144	0.362
Sr–Zn	0.290	0.026	0.037	0.508

Ti–V	0.114	0.388	−0.146	0.360
Ti–Zn	0.237	0.070	−0.020	0.465
V–Zn	0.149	0.260	−0.111	0.390
